# Nii Kandis (Knowing Myself): Finding a Sacred Home at Anishnawbe Health Toronto Through Spirit-Based Healing

**DOI:** 10.3390/ijerph23030405

**Published:** 2026-03-23

**Authors:** Allison Reeves, Anishnawbe Health Toronto, Teresa Beaulieu, Kimberly Jordon

**Affiliations:** 1 Department of Psychology, University of Guelph, Guelph, ON N1G 2W1, Canada; 2 University of Guelph-Humber, Toronto, ON M9W 5L7, Canada; 3 Indigenous Community Healthcare Centre, Toronto, ON M5A OX9, Canada; mmilward@aht.ca; 4 Weaving Wellness, Toronto, ON M6S 4W4, Canada; tera.beaulieu@weavingwellness.ca (T.B.); kimberly.jordon@weavingwellness.ca (K.J.)

**Keywords:** indigenous healing, indigenous spirituality, indigenous mental health, cultural safety, qualitative research methods, indigenous research methods, indigenous cultural resurgence

## Abstract

**Highlights:**

**Public health relevance—How does this work relate to a public health issue?**

- Research has indicated that there is a higher dropout rate amongst Indigenous clients using Western mental health treatments in North America, as Western services are not always culturally safe for Indigenous clients.
- Given the history of intergenerational and ongoing systemic trauma facing Indigenous Nations, this results in a higher-needs population being underserved by health services.

**Public health significance—Why is this work of significance to public health?**

- In recent years, Canadian healthcare services have sought to integrate Indigenous worldviews into programming, to address systemic racism in healthcare, and to promote cultural safety in healthcare.
- Our community partner, Anishnawbe Health Toronto (AHT), is Canada’s largest multidisciplinary Indigenous health centre. This project investigates cultural resurgence efforts in AHT health programming and details Indigenous-informed models and understandings of mental health.

**Public health implications—What are the key implications or messages for practitioners, policy makers and/or researchers in public health?**

- Findings detail the unique healing relationships in mental health services at AHT (“extended family model”, minimizing power imbalances, promoting client self-determination), the sacred space offered within the centre, and the role of spirituality in mental health healing and community wellness.
- These descriptions offer a model for other health centres serving Indigenous populations in North America and can inform policy makers around how spending dollars can support Indigenous mental health services.

**Abstract:**

Anishnawbe Health Toronto (AHT) is Canada’s largest multidisciplinary Indigenous health centre. In 2023, the Executive Director of AHT spearheaded a community-centered research study looking at mental and spiritual health for its community of service users. This project sought to support cultural resurgence efforts in AHT health programming through the synthesis of Indigenous-informed models and understandings of mental health, rooted in the knowledge and experience of care providers at AHT. This project also sought to enhance Indigenous community research capacity by involving Indigenous community stakeholders in each stage of the qualitative research process. This paper details these methods, which follow Indigenous community ethics in research, and include both Indigenous approaches to research as well as qualitative methods. This paper then presents a summary of the study’s findings, describing the interdisciplinary mental health services of a team of Indigenous and non-Indigenous practitioners at AHT. Three major themes describe the unique features of these services: The Healing Relationship, Indigenous Spaces and Identities as a “Sacred Home”, and Healing Through Spirit. The connection between spirituality and Indigenous wellness is discussed by centering Indigenous values and ways of knowing as central to Indigenous healing, survivance, and cultural resurgence.

## 1. Introduction

### 1.1. Spirituality and Mental Health

The relationship between spirituality and mental health has received increasing attention across psychology, psychiatry, social work, and public health disciplines [1,2,3,4]. In recent decades, researchers have sought to understand whether and how spiritual beliefs, practices, and attitudes influence mental health outcomes, coping processes, and mental health recovery trajectories [5]. Scholars note that spirituality can encompass religious beliefs, existential meaning-making, transpersonal experiences, ethical values, and practices such as ceremony, prayer or meditation, and that it can exist independently of formal religious affiliation [6,7]. In the 2025 research of Hinterberger and Walter [8], spirituality is identified as a multidimensional construct, with components of transcendence, connection to self, and spiritual attitudes (e.g., stillness, trust in life, connection to a greater whole), among others. While evidence suggests that spirituality may function as a protective factor for mental well-being [8], the construct itself remains contested, multifaceted, and culturally situated [8]. Given the wide conceptual diversity around spirituality, completing larger empirical research studies looking at the impacts of spirituality on mental health is challenging to undertake as lived spiritual experience is complex, nuanced, and personal [5,8]. Authors suggest that a more granular understanding of spiritual function in psychosocial contexts is needed to better understand its impact on mental health outcomes in specific individuals or communities [8].

Spirituality is often a central focus in traditional Indigenous healing practices around the globe [9,10], offering a sense of meaning and purpose in individual and community life [9]. Practicing spirituality-based healing both for addressing illness, as well as for ongoing health promotion, has been an aspect of daily life for many Indigenous Nations [11]. Culturally situated spirituality emphasizes connection with community, the natural world, and the spiritual world (the realm of Ancestor Spirits, spirit guides, and Creator) [10]. For Indigenous Peoples, spiritual practice and ritual provide cultural continuity, helping to address cultural dislocation related to colonial harms and intergenerational trauma [10,12,13], which continue to impact Indigenous communities in Canada and globally [13,14].

Research indicates that Indigenous Peoples engaged in mainstream mental health treatment have higher dropout rates across the globe, due to cultural incongruence, systemic racism, and a lack of cultural safety in services [15,16,17,18,19]. Western mental health training programs often overlook spirituality. In turn, Indigenous clients are less likely to engage with services that are not aligned with their values and worldviews, which have traditionally centered spirituality [10,13,20]. In recent years, Canadian healthcare services have turned their attention to consider whether programs and services for Indigenous clients are Indigenous-informed and culturally safe for Indigenous community members [21,22]. Services have sought to integrate Indigenous worldviews into programming, to address systemic racism in healthcare, and to minimize power imbalances between clients and practitioners [12,21,22].

Increasing culturally safe practice in healthcare is a step toward decolonizing Western mental health services, which continue to be rooted in settler-colonial models of health in contexts such as Canada, the United States, Australia, New Zealand, and elsewhere [23]. Western wellness models often do not focus on holistic approaches to care and instead follow a biomedical and pathology-based model [24]. Alternatively, Indigenous ways of knowing in healthcare have traditionally centered Healers, spirituality, and ceremony in wellness practices. Indigenous models are typically rooted in community and spiritual frameworks that focus on relationship with land, Ancestors, non-human relations, and all of creation [25]. Practices that centre nuanced cultural and spiritual knowledge can be emotionally therapeutic for Indigenous participants and can function to strengthen identity and reconnection to cultural knowledge that was weakened through colonialism, but that is now seeing a resurgence [25].

### 1.2. Anishnawbe Health Toronto: An Indigenous Health Centre

From 2023 to 2024, Joseph Hester, the (now late) Executive Director of Canada’s largest multidisciplinary Indigenous health centre, Anishnawbe Health Toronto (AHT), spearheaded a community-centered research study funded through a Toronto Region Health Grant looking at mental and spiritual health for its community of service users. The centre is funded through local, provincial and federal governments in Canada, and thus is subject to governmental oversight and to certain reporting requirements. Given these funding and reporting constraints, the research steering committee, comprising two Indigenous leaders at AHT, hoped to better understand how service provision maintains Indigenous approaches to healing, in spite of these Western structures and influences.

In partnership with Allison Reeves (University of Guelph), a psychology professor and clinical psychologist practicing at AHT, and Teresa Beaulieu, an Indigenous psychotherapist and director of Weaving Wellness Centre, this project sought to support cultural resurgence efforts in AHT health programming through the synthesis of Indigenous-informed models and understandings of mental health, rooted in the experiences of care providers at AHT. This project also sought to enhance Indigenous community research capacity by involving Indigenous community stakeholders in each stage of the qualitative research process. An intention of this study involved the explicit desire to ensure space for discussions on spirituality, Spirit, and sacred aspects of mental health and healing services. These topics have often received little attention in Western-based mental health programming but have been central to Indigenous wellness practices for centuries [22].

Driven by community research priorities, this project sought to explore the following qualitative research question: What are wise practices in mental health provision for Indigenous clients at AHT? Keeping in mind the considerable diversity of peoples, languages, and cultures within and between Indigenous Nations in North America, this study was grounded in the perspective of practitioners at AHT, both Indigenous and non-Indigenous, who serve First Nations, Inuit, and Métis peoples in the Toronto area, as well as others in diverse Indigenous communities who have come to reside in Toronto.

In considering wise Indigenous practices in mental health provision, the hope of the research was to use the data from this project to understand how mental health programs and services at AHT follow Indigenous values and protocol, and to improve programs, as needed. This paper describes the results from the data collection phase of this undertaking, including suggestions for programmatic improvements, and does not present an evaluation of the updated interventions themselves. It is important to note that this project was not intended to be objective, as with other empirical research; the methodological descriptions here underline the intentionality and outcome-driven focus of this type of community-based research.

## 2. Research Methods

### 2.1. Indigenous Cultural Safety in Research

The concept of cultural safety, which includes centering decolonizing values, addressing power imbalances, and reflecting on Western biases and assumptions [12], extends to healthcare programming (content and delivery) as well as to research conducted with Indigenous Peoples. In Canada, the Tri-Council Policy Statement on Ethical Conduct for Research Involving Humans [26] includes special considerations and guidelines for ethical research conducted with Indigenous Nations and communities. These guidelines emphasize the creation of “reciprocal, trusting relationships” [26] (Chapter 9) between academic researchers and the Indigenous communities with whom they conduct research. The guidelines emphasize Indigenous community engagement in setting research priorities, adherence to Indigenous governance structures in decision-making around research, and respect for community customs, among other values and standards.

The primary research partner (AR) was a natural fit for this project: Although she is a non-Indigenous academic, she had been working with AHT clinically, and directly with the Executive Director on various research projects, for over a decade, weaving Indigenous worldviews into clinical and research practice. Her co-researcher (TB) is an Indigenous psychotherapist and Director of an Indigenous mental health centre. This project was rooted in long-standing relationships between the researchers and the community, it followed a community-driven research question, and it adhered to community protocols throughout the life of the project. In these ways, this project sought to adhere to Indigenous cultural safety principles in research.

Once the project was conceptually developed, and community ethics followed, research ethics approval was sought by the academic partner’s university institution. In addition to the primary Research Ethics Board application, the University of Guelph has an additional supplementary application that must be completed when the research involves Indigenous communities, ensuring that local Indigenous codes of conduct in research are adhered to, that the research is relevant to Indigenous community needs and priorities, and that Indigenous Elders are invited as research partners, or to provide guidance.

While this project followed qualitative methods in data collection protocols (narrative interviews), it wove Indigenous protocols into the methodology. For instance, the smudging ceremony (burning sacred plants for purification of the researcher/participant, to ground the interview space, and to set healing intentions for the interview), the sharing of loose tobacco for prayer, exchanging small gifts, and sharing meals (between researcher and participant), were all aspects of the interview process. In addition, the project began with a naming ceremony. A community Elder, Talking Fox, was gifted the Anishnawbe name for this project, “Nii Kandis” (Knowing Myself) by Spirits through ceremony. While the details of this ceremony were asked to be kept private, Talking Fox explained that the significance of the ceremony was to “give life” to the project and to ensure the project remained true to its intention to bring healing to the community.

### 2.2. Narrative Methods

Qualitative methods were appropriate for this study as they allow for the exploration of the nuances of inner and community experience, while also recognizing that relationship development between participant and researcher is a natural aspect of the research interview process [27,28]. The centering of the relationship is also consistent with Indigenous values and research protocol [27,29]. This project invited participants to engage in narrative interviews. Narrative interviews are an important method in qualitative psychological research as they invite people to share local and specific accounts that contribute to a collective story for a given community of people around a topic of interest [28,29,30]. The sharing of one’s story can be therapeutic for participants in the facilitation of self-discovery, meaning making, and, in this case, Indigenous cultural resurgence [10,31].

This study invited AHT staff involved directly or indirectly in mental health service provision to participate in an interview. AHT offers a variety of interdisciplinary health services, including traditional medicine services (Healer, Elder, and Knowledge Keeper services), primary care services (physician, nursing, nurse practitioner, physiotherapy, health promotion, and dental services) as well as mental health services (psychology, psychiatry, counselling, and youth worker services), social support programs (addiction services, housing services), and others. An email offering a general overview of the study was sent to all staff members, including details on the study as well as the inclusion criteria (anyone providing more than cursory mental health supports to clients was invited to participate). Interested staff members could email the research team to ask for additional information or to schedule an interview. Interview questions were shared at this stage of the correspondence, as well as the detailed consent form.

Thirty staff members, comprising Elders and Healers, mental health practitioners, and primary care practitioners (including physicians and nurses), volunteered to participate in a 60 min audio-recorded interview. These individuals self-selected to participate, not based solely on professional designation, but also based on their view that they offered enough mental health supports to clients to adequately respond to the interview questions. These staff identified as both Indigenous and non-Indigenous practitioners, though all had comprehensive knowledge of how socio-historical factors (e.g., colonialism, systemic racism) continue to impact Indigenous Peoples’ mental, physical, emotional, and spiritual health. The majority of these respondents (22) worked in a direct mental health role (including Indigenous Healers (2), Indigenous Elders (2), psychiatrists and psychologists (4), child and youth workers (3), traditional Indigenous counsellors (4) and mental health counsellors (7)). The remaining respondents (8) worked in a primary care capacity. In total, 17 participants identified as Indigenous and 13 identified as non-Indigenous.

Interview questions were grounded in the broad research question detailed above, and asked participants for examples of how they work with clients in their practice (i.e., approaches to care), how they understand mental health phenomena through an Indigenous lens, and their notions of what practices were effective with clients. The intention of using a qualitative approach to data collection was not to derive an empirically grounded formulation of what approaches lead to certain health outcomes; rather, we were seeking to weave together a shared pool of wisdom around our question of interest.

Once audio-recorded interviews were transcribed and anonymized by the research team, qualitative data analysis took place. This involved a line-by-line review of transcripts for emergent themes by two members of the research team. In this sense, themes emerged through the interpretation of the raw data (transcripts) by the research team and connections between thematic categories were made for conceptual similarities and differences across all interview data. Transcripts were also reviewed for instances that either support or contradict the themes developed.

A community-based analysis model then took place, wherein these preliminary thematic findings were reviewed with the Indigenous steering committee, and the preliminary themes were discussed, broadened, and altered based on the steering committee’s view of what was significant in the data. For instance, the steering committee re-interpreted participant quotes in some cases and changed some thematic categories. Here, the community steering committee was able to derive its own meanings from the data in ways that were locally relevant, grounded in community knowledge, and of practical use to the agency.

## 3. Results

Driven by community research priorities, this project sought to explore ‘wise practices’ in mental health provision for Indigenous clients. The three qualitative themes representing the main findings of the project are described in Section 3: (1) The Healing Relationship; (2) Indigenous Spaces and Identities as a ‘Sacred Home’; and (3) Healing Through Spirit.

(1) The Healing Relationship

Practitioners at AHT noted the importance of getting to know the “whole person” (Indigenous Counsellor) by listening deeply to their client’s story outside of their “presenting clinical issue.” This was described as a means of forming genuine connections with clients and creating safety in the relationship. Participants spoke overwhelmingly about the healing power of relationship, and in particular, the relationship between client and practitioner. “The principle of Indigenous health…is that we’re all relations. And so, relation is an entity that heals,” noted one Primary Care Provider. Respect was a key theme in relationship building with clients, with practitioners emphasizing the importance of adopting a stance of humility. One participant shared:

*I take clients at face value. I listen to what they’re bringing to my office on that day, I see them as a person, someone in need, knowing that the shoe could easily be reversed. I could easily be walking into their position. And understanding the interconnectivity of them being in my office and the impact that we’re going to have on each other and the support that we can give for each other*.

(Primary Care Provider)

Here, this practitioner emphasizes the bi-directional nature of the healing relationship, demonstrating respect and dignity for their client, as well as personal humility.

Cultivating closeness and warmth with clients through simple but meaningful activities such as making tea, sharing a joke, offering an opening smudge (a purification ceremony through burning sacred plants), and getting to know the client’s ancestral roots, were offered as examples. Practitioners described having “authentic care” (Healer) for their clients, and shared that they took a non-expert stance to “walk alongside clients” (Mental Health Practitioner).

Clients have faced historical and ongoing harms by colonial institutions, including Western healthcare centers, which have created mistrust in some cases [17,18,19]. For this reason, practitioners acknowledged that success begins with clients simply accessing and maintaining a connection to services at AHT. One participant shared, “People showing up and walking through the door, to me, is a success story” (Primary Care Provider). Another therapist noted the importance of building rapport: “I know that that piece takes longer, I think, than the Western way, because many Indigenous Peoples have been let down so much by people in the past” (Mental Health Practitioner).

Collaborative work also entailed upholding client self-determination. Participants spoke of their work being informed by their clients’ needs, and more broadly, driven by the community’s needs. One participant shared, “If I’m listening and attentive, clients tell me how to take care of them. And from that, I’m able to have the flexibility to care for them in the way that they’re asking.” (Primary Care Provider). One participant wove these central themes into their description of building relationships in therapy:

*What I feel is core to me as a practitioner, to doing good work with people, is to focus on relationality, on an authentic caring relationship, and on supporting self-determination, and personal sovereignty. You know, people in their lives being able to make choices and to direct who they are in the world. Their choices, their moral compass. I also believe that there is something bigger that we’re all part of—we don’t know what language that is—but it’s kind of, there’s something of Creator, of Spirit, that is part of the work*.

(Mental Health Practitioner)

Here, this quote draws together the unique facets of relationship in an Indigenous mental health context, as this practitioner sees it, including authenticity, self-determination, and Spirit, as part of the core bond.

(2) AHT as a “Sacred Home”: Connecting to Indigenous Spaces and Identities

Beyond the therapeutic relationship, practitioners emphasized the healing nature of AHT as a communal gathering space and place for cultural connection. Participants described AHT as more than just a health centre: They shared that it is a meeting place for friends, community, and those who feel “like relatives.” One Mental Health Practitioner discussed the importance of living in community as central to Indigenous Ways of Being, as a sort of extended family model. Another participant stated, “That is one of the things that I like about AHT: Our clients are all connected with everybody. You are part of a big family. So, it’s not like they only come in to see me. They always come to see somebody else, too. We can support them in many different ways.” (Primary Care Practitioner)

Like a family, AHT was described as a community unto itself, with its own values system, highlighted in plaques throughout the buildings that describe AHT’s values, or in Indigenous art and murals at the centre. They spoke of AHT as a “sacred home” (Mental Health Counsellor) and community space which gives clients opportunities to invest in relationship building *on site*, and thus a place to alleviate isolation. In addition, having an environment where clients can identify with staff and other community members through their shared identity and lived experience, fosters a sense of belonging and reinforces a positive affiliation with one’s Indigenous identity. One Mental Health Practitioner noted the psychological benefits for clients to see that “the first person I see at reception looks like me”. Another participant described the significance of cultural connection at AHT: “That’s a really core value. Reconnection to our community, reconnection to our food, reconnection to our way of life.” (Primary Care Practitioner)

Notably, the spiritual aspects of AHT were emphasized as being different from mainstream health centers. One Mental Health Practitioner described AHT as a “healing container”, stating, “Spirit is in the building; this helps guide the work”. AHT’s offerings of on-site ceremonial space, including a Sweat Lodge and Shake Tent, were acknowledged as being unique in an urban centre like Toronto, and an integral part of healing through cultural connection. Practitioners also spoke of the importance of offering clients the opportunity for smudge and prayer in all of the offices and throughout the building. Connecting to the Traditional Healing team on site also represented a spiritual opportunity: Practitioners could provide a “warm transfer” (referral) to Healers just down the hall (Mental Health Counsellor). One Primary Care Provider discussed the spiritual opportunity in such an in-house transfer: “We see there is a disconnection [related to client] identity, because of the years of colonization. There’s a disconnection between who they are and their culture. So that’s where the Traditional Healer comes into play: To incorporate this. It promotes client general well-being—not just from a mental health perspective, but also for their spiritual health”.

Practitioners noted that clients can connect and/or reconnect with their Indigenous identity through AHT’s cultural programming, in addition to direct mental health services. One participant spoke of the impacts of cultural connection and decolonization efforts through a nutrition program at the centre: “[Preparing traditional food] might be something so small, but it really does make a difference in this whole process of decolonizing these spaces, and also helping the healing journey” (Primary Care Practitioner). Other participants encouraged active client engagement in community and cultural activities at AHT, including ceremonies, drumming, dancing, and beading. Pointing to her large medicine wheel poster in her office, one Traditional Counsellor described her workshop on balance: “I’m always going back to what we were as a Nation before colonization impacted us: What our values were, what the spirit of community was like…and understanding the medicine wheel”. This Traditional Counsellor acknowledged that positive identity teachings are still needed to address internalized shame around Indigenous identity as a result of long-standing racist colonial beliefs. As a counsellor, she saw her role as a “midwife for identity” who could help support clients to “wake up” their Indigenous identities.

Overall, connecting with one’s identity in the AHT community through counselling, ceremony, cultural activities, and even just being in the building itself, was described as a central facet of client healing journeys. A mental health practitioner summarized the value of reclaiming identity as part of decolonizing and healing at AHT: “That’s one of the main differentials working in a mainstream versus an Indigenous health centre: The focus on identity work. Because without that piece intact, without a secure tether to who I am and where I come from, then wholeness, wellness, and healing are elusive for most people, if not impossible. If I don’t know how I am, or who I am, how can I be well?”

(3) Healing Through Spirit

The healing approach that AHT Healers engage in is unique among all other health practitioners at the agency due to their intentional and direct work with Spirit (referring to Creator, Spirit Helpers, or Spirit Ancestors). This is not to say that other practitioners do not derive inspiration or support through Spirit and spirituality. However, of all the providers interviewed, it was only the Healers who noted that within their helping work, they offer a direct conduit for Spirit to do its healing work. In this sense, the Healers noted that they themselves do not determine the course of action in their healing work. They confer with Spirit, who directs them accordingly. They make a request of Spirit to enter the room, to assess the client’s health need, and to set a treatment path accordingly. This treatment path may involve ongoing consultation and counselling with the Healers themselves, it may involve a prescription for ceremony, a herbal remedy or other doctoring, or it may involve referral to other practitioners. It was this direct channel with Spirit that made the Healer’s work unique among other practitioners. Participants shared that this kind of healing is at the heart of AHT’s mental health services. One Mental Health Counsellor spoke of the Indigenous Healers as the core of the organization: “The Traditional People, they’re at the heart of this place. And if it was not for them, there would be no need for us to exist, frankly, because we wouldn’t be any different from any other community centre.”

More generally, participants described the unique nature of AHT’s core values as being a ‘Spirit first’ principle: “Spirit is where we are. It is who we are.” (Mental Health Practitioner). An Elder shared, “We are born into wellbeing as Spiritual beings.” Practitioners noted that their care is rooted in a spiritual understanding that there is something bigger than us as individuals, and that something greater than us is in the room: “Creator is with us”, noted one Mental Health Practitioner. Therapists spoke about their openness to bringing spiritual material into the therapy session, in the form of discussions about dreams, ancestors, spirit helpers, and spiritual beliefs. While a discussion on this type of spiritual content may be overlooked in some mainstream settings due to the lack of focus on spirituality in Western counselling, there is a central place for these discussions at AHT. One Traditional Counsellor acknowledged that while not every client is interested in Indigenous spirituality, every client should, at a minimum, know their peoples’ deep spiritual history.

Participants described unique experiential learning opportunities for clients at AHT through traditional healing. They shared that Indigenous healing offerings include naming ceremonies, where clients learn from Healers about their Spirit name, their sacred animals, their clan, and their colours. Clients can also engage in other ceremonies at AHT, including the sweat lodge ceremony, the shake tent ceremony, fasting ceremonies, and full moon and sunrise ceremonies, among others. Clients can smudge in the therapy space, go outside to lay down tobacco on the land in prayer, as well as use other tools for grounding, such as engaging in a meditative practice while on the land. Clients can utilize herbal medicines issued by the Healers, and they can engage in drumming and singing practices for connecting spiritually and for finding internal harmony and peace.

## 4. Discussion

From the perspective of AHT practitioners, an Indigenous orientation to healing involves Spirit and spirituality as a central facet of wellness, as well as deep relationships with Traditional Helpers, community, and creation. Participants described “waking up” Indigenous identities to connect with themselves, their people, and their history, which connected directly to the Spirit name of this project, Nii Kandis (Knowing Myself). Participants also shared the importance of finding “healing containers” in people, places, and sacred spaces, which were all described as integral aspects of culturally safe care. The synergistic relationship between culturally safe care, spirituality, and the development and expression of positive Indigenous identity is important to note. Many Indigenous peoples withheld, suppressed or lost aspects of identity and spiritual practices due to colonial oppression and the introduction of strategic legislation, which would see the elimination of Indigenous identity and ways of being across generations [32]. This has left some Indigenous peoples feeling uncertain, or unsafe, to identify in ways that are aligned with their ancestral lineages, for fear of ongoing persecution, questioning, or invalidation of their identities within healthcare spaces [33]. Where cultural safety within a healthcare setting exists, such as a deepened understanding of the nuances of First Nations, Métis and Inuit histories and cultures, Indigenous staff and clients can benefit from outwardly identifying and expressing identity, which further supports the emergence of cultural and spiritual practices as part of healing experiences and trajectories.

Turning our attention from identity to spirituality, this section will consider the nuances of spirituality as it relates to the community context at Anishnawbe Health. Participants in this study spoke to the central role of spirituality as a guidepost in Indigenous counselling and emotional healing. The idea that spirituality connects us back to culture, teaches us about who we are, and simply makes us feel better emotionally was broadly shared by participants across disciplines. Spirituality was described as being an internally and relationally experienced, meaning-oriented force, rather than relating to an externally imposed set of rules or regulations, as with some formal religions. Notably, the manner in which Healers and others spoke of spirituality in this study moved beyond the individual client experience to consider Spirit as its own force, existing independently of people, but of being intricately tied to all of creation. Difficult to describe in words, Healers shared about Spirit working through them to instruct them as to what healing actions (e.g., ceremony, doctoring, client behavioural change) were needed for the client in the material world. What might be described as a sense, as in hearing, seeing, or tasting, Healers described having a sense of Spirit, through a direct channel with the Spirit world. While the psychological literature might describe the benefits of spiritual belief and practice in terms of individual benefits, for instance, through improved emotion regulation, through positive attachment relationships to the concept of a Creator, through shared relational experience in group ceremony, or through physiological stress reduction, Healers in this study would likely argue that spirituality is not simply the sum of these parts. They described Spirit as a force unto itself, outside of us, benevolent in nature, that humans may seek access to, and which provides wisdom and healing powers through its own force or energies, as with a God.

For Healers, Elders, Indigenous counsellors, and other practitioners who take this perspective, there is an understandable reverence for such a spiritual power or force, and these individuals seek to protect and respect this sacred knowledge, with an understanding that in the not-too-distant past, there was wider community knowledge of Spirit amongst Indigenous Nations. Individual “mental health” was a construct that was seen as being inextricably tied to community health as well as to collective spiritual beliefs and practices. Participants in this study shared that they belong to a people who felt they already had the necessary ingredients for individual and community wellness, including the spiritual realm and spiritual ceremony as part of living well for daily health promotion. As one Elder noted, “We are born into wellbeing as Spiritual beings”. These ways of being and systems of understanding were profoundly disrupted by colonialism through forced assimilation into Euro-Western cultures and cultural genocide [10,34]. A reorientation toward these ancient wisdoms in the present day is what drives these practitioners as part of their helping work.

In their efforts to weave together traditional beliefs and practices into contemporary healthcare practice, these practitioners were committed to keeping Spirit alive, quite literally. This reclamation of traditional forms of spirituality, coupled with the innovation of integrating ancestral knowledge systems and practices within Western healthcare spaces, continues to emerge across health and healing spaces across Canada as part of a decolonizing practice in mental health provision [35,36]. Clare et al. (2025) identified that community leadership and ownership, cultural and contextual factors (e.g., interrelationships and cultural practices), workforce capacity and capability (including delivering culturally safe care), and values-based service delivery are some of the key factors for successful integrated healthcare models for Indigenous peoples [37]. The results of this study illustrate how an interdisciplinary team with Indigenous staff and leadership, who are grounded in values of relationship and spirituality, alongside non-Indigenous practitioners committed to cultural safety, can foster healing spaces that act like a sacred home for Indigenous individuals on their healing journeys.

In consultation with the steering committee and leadership at AHT, the findings from this study have informed mental health programming at AHT. The centre has deepened its traditional health program by broadening the ceremonies offered, by increasing the size of the Traditional Healing team, and by seeking to bring on more female Healers. The mental health team has completed additional training in Indigenous psychotherapy modalities that centre identity and spirituality in healing. There have been more formalized and informal cross-referrals to the healing team and the other mental health practitioners, and more collaboration between these teams in serving clients from a holistic model of care.

Some challenges in care at AHT were also noted in interviews, though few enough that they did not merit their own results section. For instance, one participant spoke of the difficulty in maintaining healthy clinical boundaries when working in a small community setting where dual (or multiple) relationships may exist; one individual named the need to recruit more female Healers at the centre, recognizing that women played a central role as Medicine People historically; and one participant acknowledged that some Indigenous clients are not interested in taking a spiritual path in their care, and prefer to access only Western services. The latter comment was not noted to be an issue, per se, but was shared as an acknowledgement of difference amongst service users. Keeping these considerations in mind, the overarching message of participants in this study continued to be an emphasis on spiritual wellbeing and spiritual growth as central to health promotion and mental wellness in this population of service users.

## 5. Conclusions

This paper presents a summary of the interdisciplinary mental health services of a team of Indigenous and non-Indigenous practitioners at an Indigenous community health centre in Toronto, Canada. Though the practitioners blend Western and traditional Indigenous approaches to care in their service delivery, what makes this health centre particularly unique is the values-driven approach to centering Indigenous clients’ ways of knowing into each healing encounter. No matter the approach, whether a visit with a Healer for a ceremony or a visit with a psychiatrist for psychotropic medication, there is always space and reverence for Indigenous beliefs, spirituality, and the presence of Spirit in care.

This study helps to identify and normalize sacred values that practitioners noted in their work, including the sense that Spirit is in the building and in ceremonial spaces at AHT, their belief in the “extended family model” of care (our words), where clients are seen as “relatives”, and their commitment to deeply listen to what their clients are telling them they need. Practitioners in this study, who understand the historical and ongoing harms of racism and Western ethnocentrism that have deeply harmed Indigenous Nations, see this extended family model of care as sacred.

A growing body of literature indicates that incorporating Indigenous healing into care, including Healer and Elder services, ceremony and cultural practices, can improve mental health outcomes and strengthen Indigenous cultural identity [10,29]. To improve equity in mental health policy, funding bodies must support Indigenous-led health centers, with appropriate and adequate funding for Indigenous healing services. This includes funding for ceremonial spaces, sacred medicines, and other components of Indigenous healing practice. It also includes paying Healers a fair, appropriate, and respectful wage. Funding dollars signal value and Healer positions have often gone underfunded [38]. Western healthcare systems are structured to recognize clinical authority rooted in professional designation, drawn from a biomedical model. Funding structures and policy makers must allow Indigenous-led governance in healthcare at the local level, so that community leadership can set standards and funding priorities, based on Indigenous values and wise practices in healing. Ideally, funding would be long-term to support sustainability in Indigenous mental health programs [39] and would recognize the value of integrated care models, with Indigenous and Western-trained practitioners working alongside one another in a hybrid model [25,35,40]. Funding for physical spaces where clients can see their culture reflected in the staff, the programming, and even the artwork increases accessibility and promotes participation in programming, as described in the present study.

Overall, efforts to decolonize mental health services are driven by both human rights considerations as well as practical considerations. From a human rights perspective, supporting Indigenous self-determination in healthcare services responds to historical and ongoing colonial harms related to racism in healthcare and the marginalization of Indigenous ways of knowing in healing. Practically, there is a growing consensus that Indigenous healing practices, including spirit-based counselling, ceremony, and working with Healers, are effective practices in supporting Indigenous mental health and wellness. These approaches can help strengthen identity, rebuild relationships within communities, and promote cultural safety in care. As research in this area continues to grow, collaborative models that respect Indigenous sovereignty and incorporate traditional healing practices are increasingly seen as important steps toward more equitable and effective mental health services for Indigenous peoples [24,25].

Future research can explore Indigenous client experiences of accessing care from culturally integrated healing programs to better understand the intersections of Western and traditional forms of care in fostering healing. This may include a particular focus on certain populations, such as Indigenous youth, families, or two-spirit community members. Exploration of other community-based Indigenous healthcare models and teams working in rural or northern contexts would illustrate potential similarities and differences across geographic regions and communities.

## Data Availability

Interview data belongs to the Anishnawbe Health Toronto community in Toronto, in keeping with ethical guidelines for research involving Indigenous peoples in Canada. (See TriCouncil Policy Statement: Ethical Conduct for Research Involving Humans). Only with community consent can this data be shared upon request.

## Abbreviation

The following abbreviations are used in this manuscript:

AHT	Anishnawbe Health Toronto
